# Identification of a Prognostic Model Based on Immune Cell Signatures in Clear Cell Renal Cell Carcinoma

**DOI:** 10.1155/2022/1727575

**Published:** 2022-08-23

**Authors:** Xuezhong Shi, Yali Niu, Yongli Yang, Nana Wang, Mengyang Yuan, Chaojun Yang, Ani Dong, Huili Zhu, Xiaocan Jia

**Affiliations:** Department of Epidemiology and Biostatistics, College of Public Health, Zhengzhou University, Zhengzhou, 450001 Henan, China

## Abstract

**Background:**

Accumulating evidence substantiated that the immune cells were intricately intertwined with the prognosis and therapy of clear cell renal cell carcinoma (ccRCC). We aimed to construct an immune cell signatures (ICS) score model to predict the prognosis of ccRCC patients and furnish guidance for finding appropriate treatment strategies.

**Methods:**

Based on The Cancer Genome Atlas (TCGA) database, the normalized enrichment score (NES) of 184 ICSf was calculated using single-sample gene set enrichment analysis (ssGSEA). An ICS score model was generated in light of univariate Cox regression and Least absolute shrinkage and selection operator (Lasso)-Cox regression, which was independently validated in ArrayExpress database. In addition, we appraised the predictive power of the model via Kaplan-Meier (K-M) curves and time-dependent receiver operating characteristic (ROC) curves. Eventually, immune infiltration, genomic alterations and immunotherapy were analyzed between high and low ICS score groups.

**Results:**

Initially, we screened 11 ICS with prognostic impact based on 515 ccRCC patients. K-M curves presented that the high ICS score group experienced a poorer prognosis (*P* < 0.001). In parallel, ROC curves revealed a satisfactory reliability of model to predict individual survival at 1, 3, and 5 years, with area under the curves (AUCs) of 0.744, 0.713, and 0.742, respectively. In addition, we revealed that the high ICS score group was characterized by increased infiltration of immune cells, strengthened BAP1 mutation frequency, and enhanced expression of immune checkpoint genes.

**Conclusion:**

The ICS score model has higher predictive power for patients' prognosis and can instruct ccRCC patients in seeking suitable treatment.

## 1. Introduction

Clear cell renal cell carcinoma (ccRCC) was a cancer with an incidence of 2.2% and a lethality of 1.8% annually according to Global Cancer Observatory reported in 2020 [1]. About 20-30% of ccRCC patients has metastasized at the time of diagnosis, with 5-year survival rates of less than 10% and weak sensitivity to radiotherapy and chemotherapy [2, 3]. Given that, it was significant to screen novel biomarkers with higher predictive value for the ccRCC patients.

The tumor microenvironment (TME) was a complex dynamic multicellular ecosystem consisting of a mixture of immune cells, stromal cells, cancer cells, and other components [4, 5]. Immune cells in the TME were long identified as a pivotal and central area of oncology investigation, performing an invaluable role in the prognosis, immune escape, and treatment resistance of malignancies [6, 7]. Dai et al. [8] postulated that intratumor CXCL13^+^ CD8^+^ T cell infiltration compromised the function of CD8^+^ T cells, rendering ccRCC patients with poor clinical outcome. Jonasch et al. [9] uncovered that the interaction of genomic instability with the TME could modulate the immune cell populations of ccRCC and in turn affected the survival of ccRCC patients, providing innovative insights for targeted therapy.

However, the majority of present studies evaluating the effect of immune cells on ccRCC were centered on minority immune cells or model construction based on immune-related genes. Wu et al. [10] identified two CD8^+^ T cell-associated molecular clusters in ccRCC to provide guidance for prognosis prediction and immunotherapy. Peng et al. [11] constructed a TME-related genes model with prognostic value using immune or stromal scores after ESTIMATE algorithm to predict patients' survival outcomes and immunotherapy responses. In addition, Gu et al. [12] developed an immune risk score model to assess the prognosis of ccRCC patients by the CIBERSORT algorithm. The CIBERSORT algorithm only counted 22 immune cells, and most immune cell signatures (ICS) were not included [13]. Consequently, there was an urgent demand to adopt a new assessment system including more ICS to predict ccRCC prognosis and guide therapy.

In this study, we utilized The Cancer Genome Atlas (TCGA)- kidney renal clear cell carcinoma (KIRC) cohort to construct an ICS score model based on 184 ICS and validated in E-MTAB-1980 cohort. Moreover, the relationships between the model and immune landscape, genetic mutations, and ailment treatment were further elaborated. This study envisaged mining prognostic indicators to assist oncologists in identifying personalized treatment strategies.

## 2. Methods

### 2.1. Data Collection and Processing

**Figure 1:**
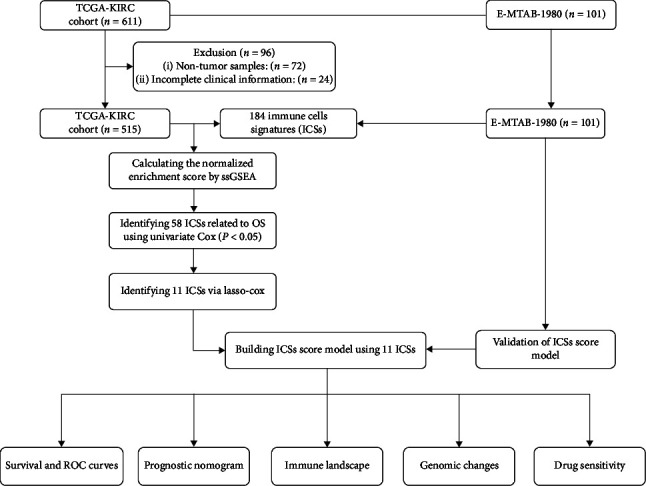
Flowchart of the study.

Figure 1 displays the flowchart of our study process. KIRC (539 tumor samples) was retrieved from TCGA database (https://portal.gdc.cancer.gov/) as training cohort. E-MTAB-1980 (101 tumor samples) was downloaded from ArrayExpress database (https://www.ebi.ac.uk/arrayexpress/) as external validation cohort. Patients were ruled out if they were not a tumor sample or did not have a complete record of clinical information (survival time, survival status, age, stage, grade, and gender) in TCGA database. The batch effect between the two cohorts was corrected using the “sva” package. For normalization, transcripts per million values were processed by log_2_ (value + 1), which were analogous to gene expression from microarrays and comparable between patients.

### 2.2. Gene Set Enrichment Analysis

A total of 184 ICS were obtained by retrieving previous literature [14]. The single-sample gene set enrichment analysis (ssGSEA) [15] was implemented to calculate the normalized enrichment score (NES) of each ICS using the “GSVA” package. The NES was considered as the infiltration levels of 184 ICS for each ccRCC patient.

### 2.3. Construction of ICS Score Model

Univariate Cox regression was performed to screen the prognosis-related signatures in the training cohort. In addition, the Least absolute shrinkage and selection operator (Lasso)-Cox regression was applied to exclude variables with a regression coefficient equal to zero, tackling the problem of overfitting. After the shrinkage via “glmnet” packages, the optimal *λ* value was acquired [16]. Ultimately, we constructed an ICS score model through the following formula:

(1) $$\begin{array}{c} \text{ICSs score}=\overset{n}{\underset{i=1}{\sum }}\text{C}\text{OE}{\text{F}}_{i}*\text{NE}{\text{S}}_{i}, \end{array}$$

where COEF referred to the regression coefficients stemming from univariate Cox regression and NES represented the NES for the corresponding ICS.

### 2.4. Assessment and Validation of Model

To evaluate the predictive performance of the model, patients in the training cohort were classified into high and low ICS score groups based on the median ICS score. Kaplan-Meier (K-M) survival curves were applied for survival comparison between the high and low ICS score groups. Time-dependent receiver operating characteristic (ROC) curves including survival at 1, 3, and 5 years were established to reflect the sensitivity and specificity of the model. Calibration curves were performed to compare the actual and predicted probability of overall survival (OS) at 1, 3, and 5 years.

### 2.5. Independent Prognostic Analysis

After initial filtering by univariate Cox analysis, multivariate Cox analysis was conducted to assess the implication of independent prognosis for grade, age, gender, stage, and ICS score variables. Following confirmation of the prognostic value of ICS score, a nomogram integrating clinical traits and the ICS score was constructed to predict the survival probability of each patient. The forecast performance of the nomogram was evaluated by the *C*-index and calibration curves.

### 2.6. Immune Infiltration Analysis

We used the ESTIMATE [17] algorithm to calculate the immune infiltration status of immune components. In the ESTIMATE algorithm, we calculated the scores using the “estimateScore” function, which calculated the matrix, immune, and estimated values for each sample based on the gene expression data, so the results were stable when the gene expression data were determined. Moreover, we utilized the CIBERSORT algorithm to estimate the abundance of 22 types of immune cells. In the CIBERSORT algorithm, we set perm = 1000, meaning that a single sample was repeated 1000 times to estimate the *P* value of immune infiltration so as to obtain stable results. And we run the two algorithms separately more than three times with the same results. Afterwards, we employed the ssGSEA to evaluate the abundance of different immune-associated functions or pathways.

### 2.7. Mutation Analysis

The somatic variant landscape was visualized by the “maftools” package [18]. Tumor mutation burden (TMB), commonly defined as the total number of nonsynonymous mutations, was proposed as a promising biomarker of immunotherapy [19]. The TMB of each patient between high and low ICS score groups was assessed.

### 2.8. Immunotherapy Forecast and Drug Sensitivity Analysis

Immunophenoscore (IPS) representing four categories of immunogenicity-determining genes (effector cells, immune suppressor cells, MHC molecules, and immune modulators) was calculated using an unbiased machine learning approach. It has been authenticated that the higher IPS score, the stronger the immunogenicity and the better the response to immunotherapy. The IPS of ccRCC patients were originated from The Cancer Immunome Database (TCIA, https://tcia.at/home) [20].

Genomics of Drug Sensitivity in Cancer (GDSC) (https://www.cancerrxgene.org/) was a publicly available pharmacogenomic database to study the drug sensitivity of cancer cells, which could present a distinct resource for the discovery of new targets for cancer therapy [21]. Half-maximal inhibitory concentrations (IC50) of common chemotherapeutic agents were estimated by the “pRRophetic” package [22].

### 2.9. Statistical Analysis

All statistical analysis was implemented using R software (version 4.1.2). Continuous variables between the two groups were compared using the Wilcoxon test. The Chi-square test and Fisher's exact test were performed to compare categorical variables. Pearson correlation test was used to infer the correlation between the two parameters. All statistical *P* values were two-sided. Unless otherwise stated, *P* < 0.05 was considered significant.

## 3. Results

### 3.1. Identification of Prognosis-Related Signatures and Construction of ICS Score Model

**Figure 2:**
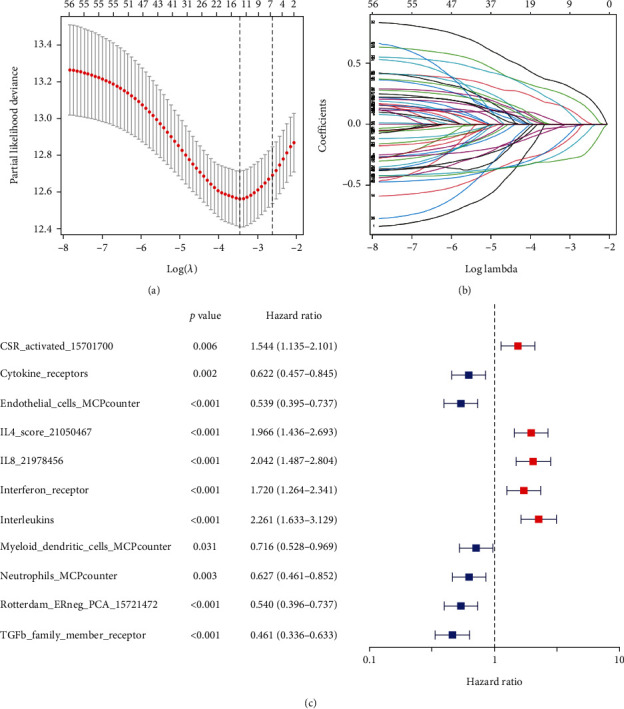
Construction of the ICS score model in the training cohort. (a) Lasso-Cox coefficient profiles of 11 selected ICSs. (b) Disclosure of partial likelihood bias. (c) Association between the 11 ICSs and OS.

The detailed demographic and clinical characteristics of ccRCC patients in this study are summarized in Table 1. A total of 515 ccRCC patients from the TCGA-KIRC cohort and 101 ccRCC patients from the E-MTAB-1980 cohort were ultimately enlisted in this study. After univariate Cox regression, 58 ICS were subjected to Lasso-Cox regression for further shrinking. Then, 11 ICS were included and used to build the ICS score model in the training cohort, when the Lasso-Cox regression reached the optimal *λ* value (0.07290335) (Figures 2(a) and 2(b)). These 11 ICS were myeloid dendritic cells MCPcounter, neutrophils MCPcounter, endothelial cells MCPcounter, cytokine receptors, interleukins, IL4 score 21050467, IL8 21978456, interferon receptor, TGF-*β* family member receptor, Rotterdam ERneg PCA 15721472, and CSR activated 15701700, of which 5 signatures were hazard factors while 6 signatures were protective factors (Figure 2(c)).

### 3.2. Evaluation and Verification of the ICS Score Model

**Figure 3:**
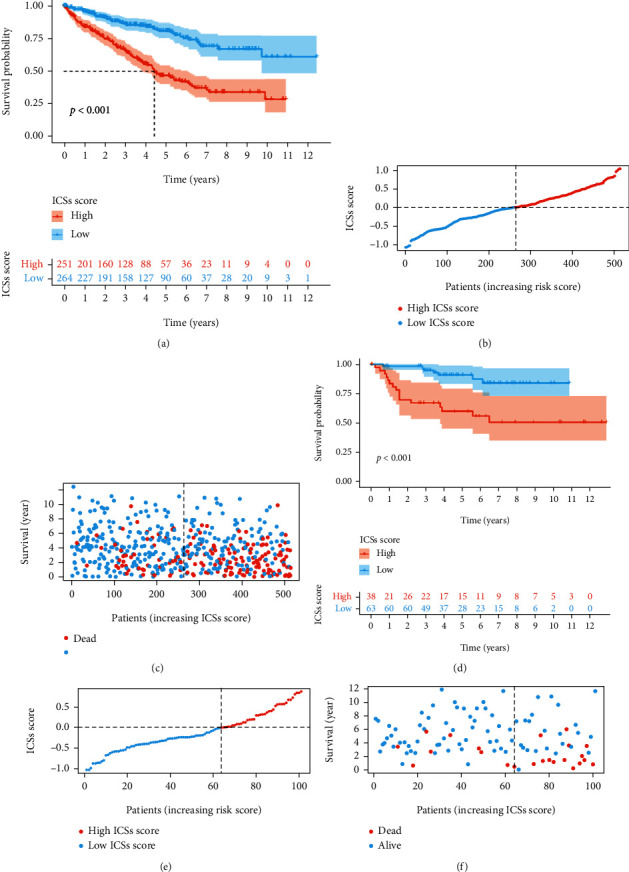
Evaluation of ICS score model. (a) Estimation of the OS in the training cohort. (b) Distribution curve in the training cohort. (c) Survival scatter diagram in the training cohort. (d) Estimation of the OS in the validation cohort. (e) Distribution curve in the validation cohort. (f) Survival scatter diagram in the validation cohort.

**Figure 4:**
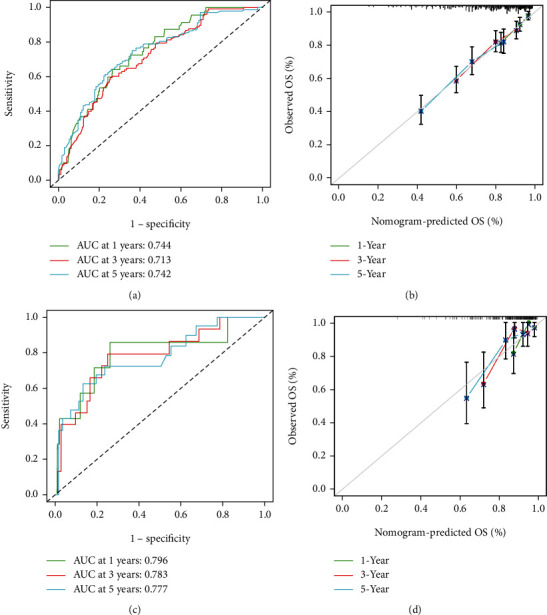
Estimation of the prognostic prediction efficiencies. (a) Time-dependent ROC analysis in the training cohort. (b) Calibration plot of the model in the training cohort. (c) Time-dependent ROC analysis in the validation cohort. (d) Calibration plot of the model in the validation cohort.

CcRCC patients were divided into high (*n* = 251) and low (*n* = 264) ICS score groups based on the median ICS score. The K-M survival curves revealed significantly favorable OS in the low ICS score group (Figure 3(a)). The distribution curve and survival scatter diagram indicated that patients with a high ICS score had a worse prognosis (Figures 3(b) and 3(c)). Meanwhile, the K-M curves, distribution curve, and survival scatter diagram in the validation cohort were identical to the results of training cohort (Figures 3(d)–3(f)). Analysis of the prognostic prediction efficiencies indicated the model had relatively high area under the curves (AUCs) at 1, 3, and 5 years which were 0.744, 0.713, and 0.742, respectively (Figure 4(a)). The calibration curves delivered a high degree of consistency between predictions and observations in the training cohort (Figure 4(b)). Analogously, the AUCs at 1, 3, and 5 years were 0.796, 0.783, and 0.777 separately and the calibration curves also implied an excellent concordance between predictions and observations in the validation cohort (Figures 4(c) and 4(d)).

### 3.3. Prognostic Impact of ICS Score Model and Establishment of the Nomogram

**Figure 5:**
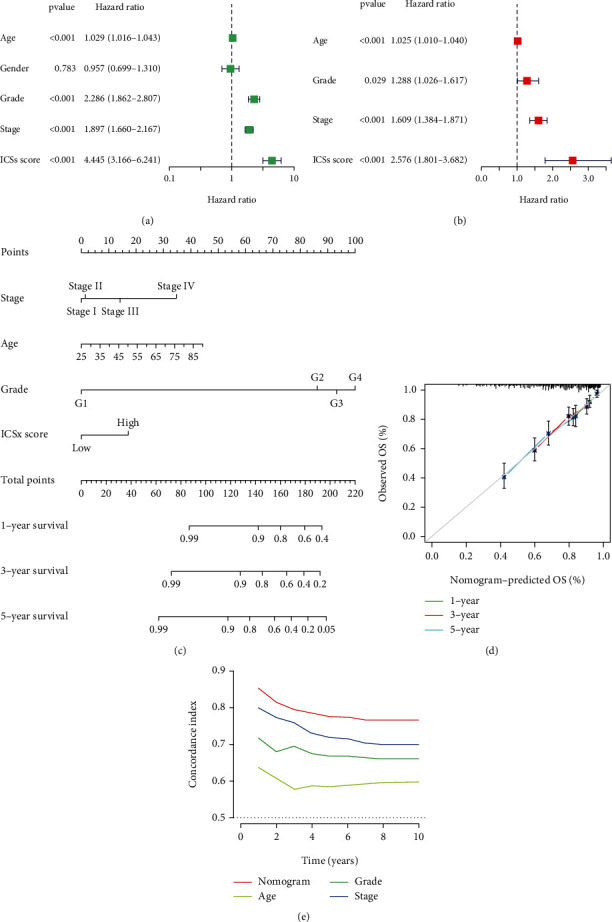
Validation of independent prognosis and construction of nomogram in the training cohort. (a, b) Univariate and multivariate Cox regression analysis of the association between clinicopathological parameters and ICS score. (c) Nomogram for predicting the probability of OS at 1, 3, and 5 years. (d) The *C*-index of the nomogram. (e) The calibration plot of the nomogram.

Univariate and multivariate Cox regression revealed the ICS score was independently correlated with OS, with a HR of 2.576 (95%CI = 1.801-3.682, *P* < 0.001), along with a HR of stage (1.609, 95%CI = 1.384-1.871, *P* < 0.001), a HR of grade (1.288, 95%CI = 1.026-1.617, *P* = 0.029), and a HR of age (1.025, 95%CI = 1.010-1.040, *P* < 0.001) (Figures 5(a) and 5(b)). To define a quantitatively individual scoring system for each patient, a nomogram that integrated the stage, grade, age, and ICS score was generated (Figure 5(c)). The *C*-index verified that nomogram manifested a satisfactory prediction in the OS of ccRCC patients (Figure 5(d)) and calibration curves testified a desirable consistency between the predicted and observed values at the odds of 1, 3, and 5 years' survival (Figure 5(e)).

### 3.4. Estimation of TME with ICS Score Model

**Figure 6:**
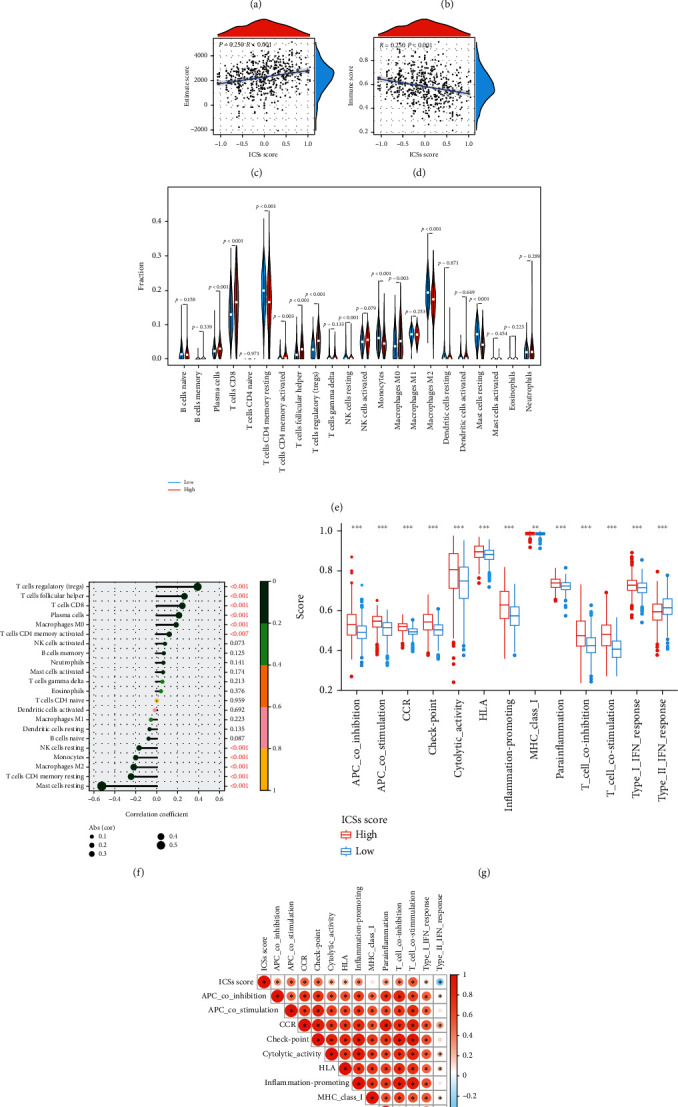
The immune status of ccRCC patients between high and low ICS score groups in the training cohort. (a) ESTIMATE analysis of StromalScore, ImmuneScore, and EstimateScore. (b–d) Correlation of ICSs score and ImmuneScore, EstimateScore, and TumorPurity. (e) CIBERSORT analysis of the relative proportions of immune infiltration for 22 immune cells. (f) CIBERSORT analysis of the correlation between immune infiltration for 22 immune cells and ICS score. (g) ssGSEA of different immune functions. (h) ssGSEA of the correlation between immune functions and ICS score. Levels of statistical significance: ^∗^*P* < 0.05, ^∗∗^*P* < 0.01, and ^∗∗∗^*P* < 0.001.

A battery of thorough analysis was conducted to appreciate the immunologic nature between the high and low ICS score groups. Initially, the result of ESTIMATE illustrated that the high ICS score group exhibited a higher ImmuneScore and EstimateScore which entailed a lower TumorPurity (Figure 6(a)). Meanwhile, there was a significant positive correlation between ICS score and ImmuneScore and EstimateScore, as well as a negative correlation between ICS score and TumorPurity (Figures 6(b)–6(d)). Subsequently, the CIBERSORT demonstrated that in the high ICS score group, the antitumor lymphocyte cell subpopulations such as CD8+ T cells, T cell CD4 memory activated, T cell regulatory, and neutrophils were significantly increased. However, the proportions of T cell CD4 memory resting, NK cell resting, monocytes M2, and mast cell resting were significantly decreased (Figure 6(e)). Further, correlation analysis verified the above results (Figure 6(f)). The ssGSEA displayed that the high ICS score group possessed a universally higher infiltration of immune functions apart from type II IFN response (Figure 6(g)). And significant correlations between ICS score and different immune functions were found (Figure 6(h)).

### 3.5. Comparison of Genomic Alterations between High and Low ICS Score Groups

**Figure 7:**
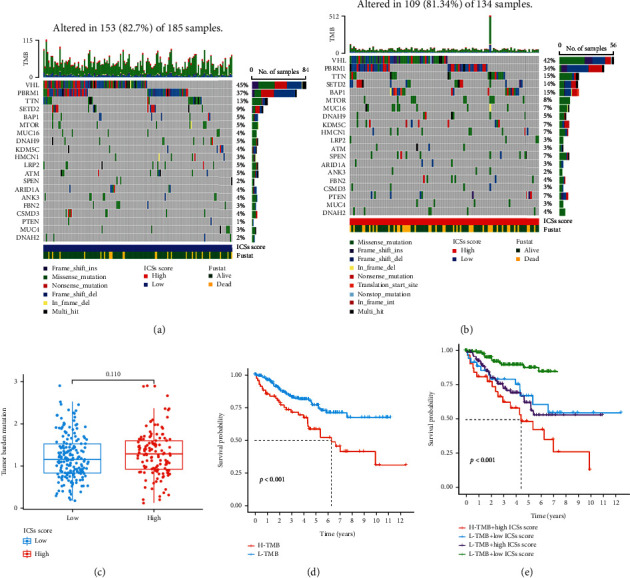
Mutation scenery in the training cohort. (a) Waterfall plot of the mutation distribution of the top 20 most frequently mutated genes in the low ICS score group. (b) Waterfall plot of the mutation distribution of the top 20 most frequently mutated genes in the high ICS score group. (c) TMB differences between high and low ICS score groups. (d) K-M curves of OS between high and low TMB groups. (e) K-M curves of OS in the combination of mutation and ICS score group.

The mutation scenery between the high and low ICS score groups was analyzed and visualized (Figures 7(a) and 7(b)). In the beginning, the mutation rates were comparable between the high and low ICS score groups (109/134, 81.34% vs. 153/185, 82.70%, *P* = 0.922). Furthermore, the five most frequently mutated genes and the most common mutation type in both high and low ICS score groups were VHL, PBRM1, TTN, SETD2, and BAP1 and missense mutation, accordingly. Subsequently, the mutation rate of BAP1 was significantly rose in the high ICS score group as shown in Table 2 (*P* = 0.004). However, there was no statistical difference of TMB between the high and low ICS score groups (Figure 7(c)). Interestingly, survival analysis noted that patients with an increased level of TMB correlated with a poor OS. And the combination of low mutation and low ICS score group had the most prolonged survival (Figures 7(d) and 7(e)).

### 3.6. Relationship between ICS Score and Treatment Strategies

**Figure 8:**
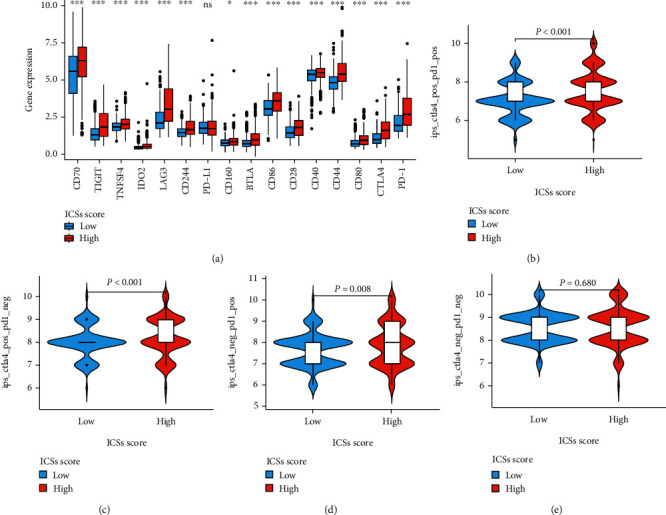
Prediction of immunotherapy response in the training cohort. (a) Differences of immune checkpoint genes between high and low ICS score groups. (b–e) Differences of IPS between high and low ICS score groups.

**Figure 9:**
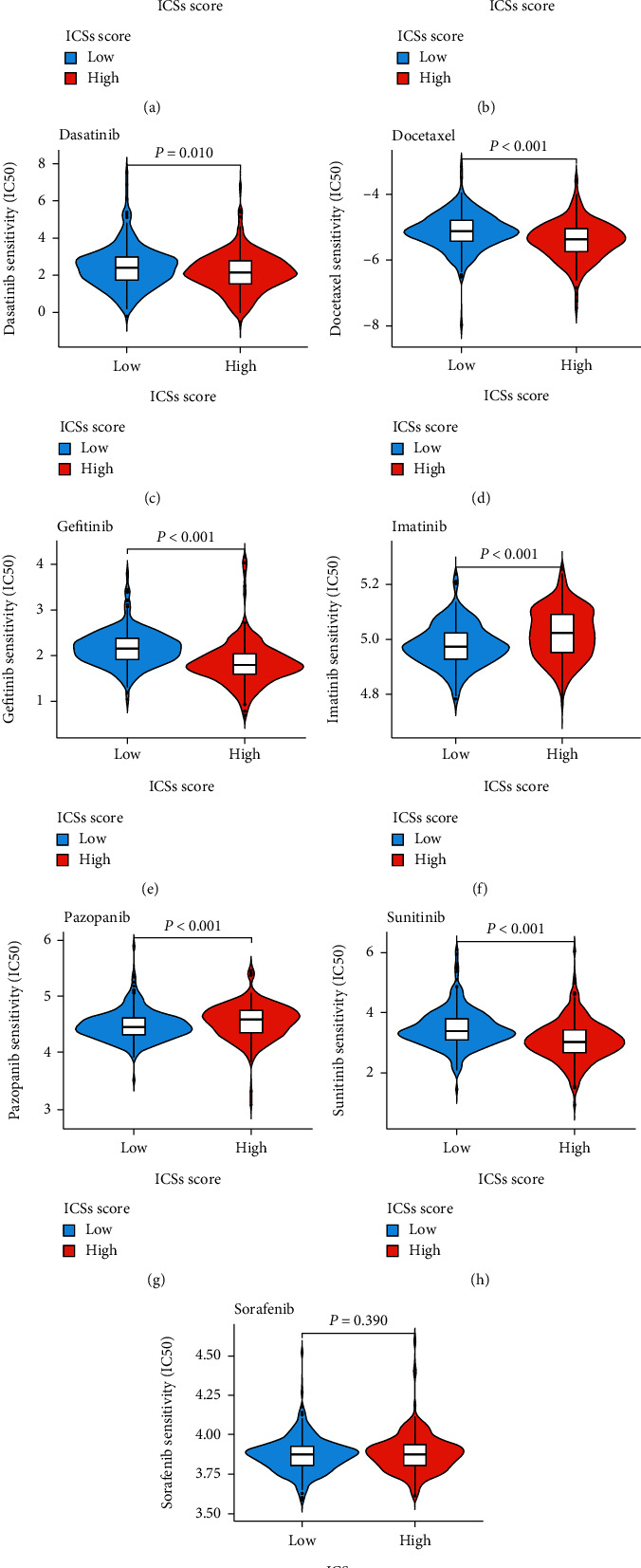
Analysis of drug sensitivity in the training cohort. (a–i) Differences in IC50 of the chemotherapeutic drugs.

To evaluate which group was more applicable for immunotherapy, several immune checkpoint genes [23] and IPS were introduced for investigation. As depicted in Figure 8(a), the expression of immune checkpoint genes was substantially upregulated in exception to programmed cell death 1 ligand 1 (PD-L1) in the high ICS score group. Meanwhile, patients with higher ICS score had significantly higher IPS cytotoxic T lymphocyte-associated antigen-4 (CTLA4)-positive-programmed cell death 1 (PD-1)-positive, IPS-CTLA4-positive-PD-1-negitive, IPS-CTLA4-negitive-PD-1-positive (Figures 8(b)–8(e)). Additionally, the data of drug susceptibility showed that the high ICS score group patients had a dramatic sensitivity to sunitinib, docetaxel, bortezomib, gefitinib, and dasatinib compared to low ICS score group patients, who exhibited a higher sensitivity to axitinib, pazopanib, and imatinib (Figures 9(a)–9(i)).

## 4. Discussion

Immune cells in the TME were essential factors affecting the progression, prognosis, and therapy of ccRCC [24]. In this study, we constructed an ICS score model based on 184 ICS. ROC curves and calibration curves corroborated the robust competence of the model to evaluate prognosis. What's more, higher ICS score was associated with more infiltration of immune cells, higher BAP1 mutation rate and better adaptation to immunotherapy. This highlighted the essential role of the ICS score model in establishing a prognostic prediction system and providing a therapeutic reference for ccRCC patients.

The ICS score embraced 11 ICS. Myeloid dendritic cells generated chemokine ligand 17 that recruited T cells as well as other activated antigen-presenting cells and was an independent prognostic factor for OS in ccRCC patients [25]. Tumor-infiltrating neutrophils were involved in the prognostic deterioration of ccRCC by releasing elastase that broke down cell-cell adhesion and promoted tumor propagation [26]. Interferon receptors, cytokine receptors and interleukins could impair the prognosis of ccRCC through reconfiguring the immune landscape of TME [27, 28]. In addition, TGF-*β* was also a member of the cytokine family. Reports claimed that the deletion of TGF-*β* receptors triggered the dysregulation of TGF-*β* signaling which further led to enhanced metastatic of ccRCC [29, 30]. Regarding the role of other ICS in ccRCC, further studies were expected to confirm. In general, these ICS had valuable effects in the occurrence, progression, prognosis, and treatment of ccRCC.

The TME were not simply consisted of the tumor cells but also the stromal cells which can be infiltrated by tumor cells and equipped with tumor-associated effects [31]. Our study observed significant difference in ImmuneScore between high and low ICS score groups, while the StromalScore was not significantly related to ICS score. Therefore, the following analysis principally recounted the immune landscape between high and low ICS score groups. It was unearthed that patients with high ICS score were evidently richer in immune cells and more likely to benefit from immunotherapy, which was inconsistent with the inferiority survival exhibited by high ICS score group patients. A substantial body of studies revealed the immunosuppressive properties of TME in ccRCC patients. Chevrier et al. [32] found that T cells and tumor-associated macrophages (TAMs) were the main immune cell populations in ccRCC. Among them, CD4CD25 regulatory T cell-suppressing T cell immunity was perceived as a principal impediment governing immunotherapy [33]. Meanwhile, high infiltration of M2-like TAMs limited the efficacy of antitumor T cell responses and exhibited high expression of HLA class II as well as complement-related genes, which were associated with tumor cells proliferation and resistance to chemotherapy [24, 34–36]. In addition, Zou et al. [37] illustrated upregulation of checkpoint molecules dampened the activity of effector T cells and antigen-presenting cells, impeding an effective antitumor immune response. Together, these contributed to explain why patients in the high ICS score group had a dismal OS despite a high infiltration of immune cells.

In a bid to furnish proper clinical treatment strategies, we would like to figure out whether the ICS score could predict the response to immunotherapy in ccRCC patients. Our study uncovered that the expression of CTLA4 and PD-1 was strikingly elevated in the high ICS score group, which was further validated by IPS, whereas no apparent differences were detected in the PD-L1 expression and TMB between high and low ICS score groups. Although PD-L1 was the most promising biomarker for predicting immunotherapy response for most malignancies, its predictive value continued to be controversial in ccRCC. Motzer et al.'s CHEKMATE-025 trial showed that irrespective of PD-L1 expression, nivolumab and everolimus improved OS in patients with refractory ccRCC consistently [38]. The inconsistency of detection methods and the variability of the thresholds used to define PD-L1 positivity were probably the most prominent factors. Meanwhile, PD-L1 expression measured by transcriptomic data was not as credible as the intensity and location of PD-L1 expression detected by immunohistochemistry. To understand whether the prognostic effect of ICS score was related to genetic alterations, we compared the disparities of genomic layer between high and low ICS score groups. It was certified that BAP1 mutation evoked augmented expression of C-C chemokine receptor 5 (CCR5) on Tregs and tumor cells. Tumor cells could secrete CCR5 ligands which bonded to CCR5, induced increased PD-L1 expression, and recruited CCR5 Tregs to local TME, thereby enhancing immune escape [39]. Extrapolating from the above results, we speculated that alterations in genes were involved in the prognosis and therapy of ccRCC patients.

There were a few of drawbacks for this study. Foremost, it was grounded in public database which deserved further validation in a prospective cohort of patients receiving immunotherapy. Moreover, integrated analysis of multiomics in the future will enable to compensate for the current deficiency of exclusive attention to data on transcriptional expression and mutation levels.

## 5. Conclusions

In summary, the ICS score model is very valuable for predicting the prognosis of ccRCC patients and is intimately interrelated with the potency of treatment. This comprehensive model has excellent prediction ability and provides appropriate individualized treatment strategies for ccRCC patients.

## Figures and Tables

**Table 1:** Clinicopathological characteristics of the ccRCC patients.

Variables	TCGA (*n* = 515)	E-MTAB-1980 (*n* = 101)
Status		
Dead	169	23
Alive	346	78
Age (years)		
≤65	340	57
>65	175	44
Gender		
Male	336	77
Female	179	24
Grade		
1	13	13
2	224	59
3	205	22
4	73	5
Undetermined	—	2
Stage		
I	257	66
II	54	10
III	122	11
IV	82	14

**Table 2:** Mutation rate of BAP1 in high and low ICS score groups.

Gene	H-wild	H-mutation	L-wild	L-mutation	*P* value
BAP1	114 (85.07%)	20 (14.93%)	176 (95.14%)	9 (4.86%)	0.004

## Data Availability

The datasets are available in TCGA database (https://portal.gdc.cancer.gov/) as well as ArrayExpress database (https://www.ebi.ac.uk/arrayexpress/).

## Abbreviations

ccRCC: Clear cell renal cell carcinoma
ICS: Immune cell signatures
TME: Tumor microenvironment
NES: Normalized enrichment score
ssGSEA: Single-sample gene set enrichment analysis
Lasso: Least absolute shrinkage and selection operator
TCGA: The Cancer Genome Atlas
TAMs: Tumor-associated macrophages
K-M: Kaplan-Meier
OS: Overall survival
Time-dependent ROC: The time-dependent receiver operating characteristic
AUCs: Area under the curves
TMB: Tumor mutation burden
IPS: Immunophenoscore
TCIA: The Cancer Immunome Database
GDSC: Genomics of Drug Sensitivity in Cancer
IC50: Half-maximal inhibitory concentrations
CTLA-4: Cytotoxic T lymphocyte-associated antigen-4
PD-1: Programmed cell death 1
PD-L1: Programmed cell death 1 ligand 1
CCR5: C-C chemokine receptor 5.
